# Sex differences in eyewitness memory: A scoping review

**DOI:** 10.3758/s13423-023-02407-x

**Published:** 2023-10-30

**Authors:** Emma M. Russell, Mitchell G. Longstaff, Heather Winskel

**Affiliations:** 1 Faculty of Health, Southern Cross University, Hogbin Dr, Coffs Harbour, NSW 2450 Australia; 2 Department of Psychology, James Cook University, Singapore, Singapore

**Keywords:** Sex differences, Eyewitness memory, Memory recall, Face identification, Facial recognition, Own-gender bias, Attention

## Abstract

Researchers in cognitive and forensic psychology have long been interested in the impact of individual differences on eyewitness memory. The sex of the eyewitness is one such factor, with a body of research spanning over 50 years that has sought to determine if and how eyewitness memory differs between males and females. This research has significant implications across the criminal justice system, particularly in the context of gendered issues such as sexual assault. However, the findings have been inconsistent, and there is still a lack of consensus across the literature. A scoping review and analysis of the literature was performed to examine the available evidence regarding whether sex differences in eyewitness memory exist, what explanations have been proposed for any differences found, and how this research has been conducted. Through a strategic search of seven databases, 22 relevant articles were found and reviewed. Results demonstrated that despite the mixed nature of the methodologies and findings, the research suggests that neither males nor females have superior performance in the total amount of accurate information reported, but rather that females may have better memory for person-related details while males may perform better for details related to the surrounding environment. There was also consistent evidence for the own-gender bias. There was some consensus that differences in selective attention between males and females may underlie these sex differences in eyewitness memory. However, none of the studies directly tested this suggested attentional factor, and thus future research is needed to investigate this using a more systematic and empirical approach.

## Introduction

Despite advances in DNA analysis, there is still a significant reliance on eyewitness memory as a critical form of evidence within the criminal justice system. Eyewitness memory is utilised in numerous ways within these settings, such as providing witness statements and information to law enforcement, identifying suspects, and giving eyewitness testimony during judicial proceedings. Errors or omissions in eyewitness memory can thus have significant implications, from confounding police investigations to the wrongful conviction of innocent people, or even to the lack of conviction of guilty people. For instance, according to data from the USA, 69% of DNA-exonerated cases were wrongfully convicted as a direct result of memory errors and misidentification by eyewitnesses (Innocence Project, 2020). Given the significance of its impact on personal and community welfare, understanding the individual differences, such as the sex or gender of the eyewitness, that may influence the accuracy of eyewitness memory is thus of continuing relevance.

Following the renewed interest in witness memory research during the 1970s, the topic of sex differences became a focus of investigation, with a sub-set of literature developing across the 1980s and 1990s in particular. The findings from this research were largely inconsistent, however, and consequently little consensus was achieved (Areh, 2011). This focus on sex or gender differences seemed to fade for a period following this with few studies published, perhaps due to the inconclusive outcomes of the earlier research. However, interest has revived in recent years, corresponding with the current salience of social issues related to gendered crime. For instance, socio-political movements, such as the #MeToo movement that rose to prominence in 2017 (Levy & Mattsson, 2020), as well as some related high-profile court cases, have brought significant attention to gendered issues such as sexual assault and domestic violence. This also extends to associated debate related to the veracity and reliability of the memories of the males and females involved. The effects of this have been seen across the world, with the number of sexual crimes reported to police increasing by 10% across 30 countries within the first 6 months of the #MeToo movement alone (Levy & Mattsson, 2020). Sexual assault and domestic violence are both heavily gendered crimes, with the majority of offences committed by male perpetrators against female victims (Tidmarsh & Hamilton, 2020; Wilcox et al., 2021). Furthermore, eyewitness testimony is often the primary evidence in investigations and court proceedings related to these crimes, especially when there is limited physical evidence or the accused perpetrator has no known history of violence (Lievore, 2004; Silva, 2022).

In the context of misidentification, as discussed above, sexual crimes are often particularly susceptible, especially when the perpetrator is a stranger to the victim or eyewitnesses (Gross et al., 2005). In fact, a review of exonerations in the USA found that of 121 rape case exonerations, 88% had involved convictions based on eyewitness identifications that had turned out to be mistaken (Gross et al., 2005). This has significant implications, particularly in light of the increased reporting of sexual crimes, which indicate that investigations and court cases related to gendered sexual violence will only increase in coming years. Furthermore, some high-profile cases that emerged from the #MeToo movement, such as Harvey Weinstein, have also highlighted the way that research regarding eyewitness memory accuracy is used – or even misused – within the criminal justice system (Conway, 2021). The prominence of these cases in recent years along with more general issues related to gendered crime could lead to broader misperceptions about gender differences in witness memory. That is, while the above examples highlight the role of witness memory and identification within gendered crime, the question of gender differences in witness memory also applies more generally to non-gendered crime. Gaining a better understanding of how sex and gender may affect eyewitness memory is therefore of ongoing relevance.

It is equally important to note that increased awareness of issues such as incomplete, inaccurate memories and misidentification does not mean that eyewitness memory should be discredited as a useful form of evidence. While the literature has demonstrated that eyewitness memory is malleable, this does not mean it is inherently unreliable, although this perception has gained traction, particularly within the legal system (Wixted et al., 2018). Disregarding eyewitness evidence can come with its own negative consequences as being disbelieved or having their memory doubted can be a source of secondary traumatisation for eyewitnesses, particularly when they are also the victim (Mason & Lodrick, 2013). Furthermore, despite rising awareness of the malleability of eyewitness memory, eyewitness testimony will remain an important form of evidence in both civil and criminal cases, including for crimes such as sexual assault. Given the implications for both the over- and under-estimation of eyewitness memory accuracy, it is therefore important to gain a better understanding of whether sex/gender does influence eyewitness memory, and the conditions under which this may have a particular effect.

### Purpose of the scoping review

Scoping reviews are a useful tool for mapping the evidence in a broad area of research in order to determine the extent of the available evidence, how the research has been conducted, and also to clarify gaps in the literature (Peters et al., 2015). Therefore, given the variability in approaches, methodologies, and findings of the literature to date, this type of review is the most suitable for our purposes. To our knowledge, no other scoping review has been conducted on this topic. The objective of this scoping review is to examine and map the range of research that has been conducted on the topic of sex differences in eyewitness memory. The specific questions for review were:

1. Are there sex differences in eyewitness memory and, if so, what are they?
2. If differences have been found, what explanations have been proposed for them?
3. What methodologies have been used to examine sex differences in this context?

## Method

A scoping review protocol was developed based on the methods outlined by the Joanna Briggs Institute Methods Manual for scoping reviews, and findings are reported in accordance with the PRISMA extension for scoping reviews (PRISMA ScR) (Tricco et al., 2018).

### Eligibility criteria

The following inclusion criteria were defined to guide the search process and decisions on the sources to be included in the review:

- Published in the English language: for the feasibility and timely completion of the review.
- Years 1970–2022: this is the period during which most of the relevant eyewitness research has been conducted.
- Adults aged 18 years and older: children’s eyewitness memory is a separate area with its own research literature and age could be a confounding factor.
- Primary research: as we are trying to map the research that has been conducted to date and how it has been conducted.

Exclusion criteria were as follows:

- Review articles (systematic reviews, meta-analyses, etc.)

### Databases

Seven databases were selected and searched for this review. These were APA PsycInfo via EBSCO, Psychology and Behavioral Sciences Collection, MEDLINE via EBSCO, Web of Science Core Collection, Proquest, Scopus, and HeinOnline. The authors, together with an experienced librarian, judged that these seven databases would be able to reach all the journals and articles relevant to the research question. By using the search terms and databases, a total of 1,424 results were found.

### Search strategy

Key terms were selected to be used in constructing search terms for each concept in order to find as many relevant results as possible (Table 1). Search strings were adjusted as appropriate for each database. Results were filtered by date range (1970–2022) where needed, and language (English). Searches in each database were documented and final results were exported to EndNote (X9) where duplicates were removed. The full search for the APA PsycInfo via EBSCO database is documented in Appendix Table 4. Reference lists of the identified papers were examined and citation searching was undertaken to identify any additional articles of relevance that were not found through database searches.

**Table 1:** Search concepts and terms used for search strategy

Concept	Keyword Search Terms
Sex	sex* OR gender OR m?n OR wom?n OR male OR female
Eyewitness	Eyewitness*
Memory	memory OR memories OR “memory recall” OR “memory retrieval” OR “memory retention” OR “memory reconstruction”
Primary research	experiment* OR study OR studies
Adults	adult* AND NOT child*

### Selection of sources

Studies for review were selected through a three-stage screening process. In the first stage, titles were reviewed to determine the eligibility according to the keywords and the defined inclusion and exclusion criteria. Abstracts were then screened, with those that were relevant to the research questions and that met the criteria selected for inclusion. Finally, the full text of each article was examined for compliance with the eligibility criteria. There is no specific process for evaluating quality outlined by the scoping review methodology, and as this is not a specific aspect of the research questions outlined for this review, full-text articles were included as long as they met the eligibility criteria and were sufficiently relevant to the research question and objectives. Three articles were excluded in this final stage as they were not able to provide information for answering the review questions for the following reasons: one included only female participants and thus did not provide information regarding sex differences; one focused on personality differences with insufficient information about sex differences; and one was not conducted in the context of eyewitness memory.

### Data charting

A data-extraction framework was developed (Table 2) and data were extracted from the articles and summarised into tables. The data-charting tables were updated continually in an iterative process. The information extracted included year of publication, country of origin/publication, population/sample size, methodology, findings related to research question, explanation/theoretical framework for findings. The information extracted was considered to be sufficient for answering the research questions.

**Table 2:** Data extraction framework

Bibliographic	Methodological	Characteristics of the article
Authors	Population / sample size	Sex differences
Year of publication	Methods used (variables, materials/measures, procedure)	Sex similarities / no sex differences
Country of origin / publication	Sex of central person/people	Explanations / theoretical framework for findings

## Results

### Selection of sources of evidence

**Fig. 1:**
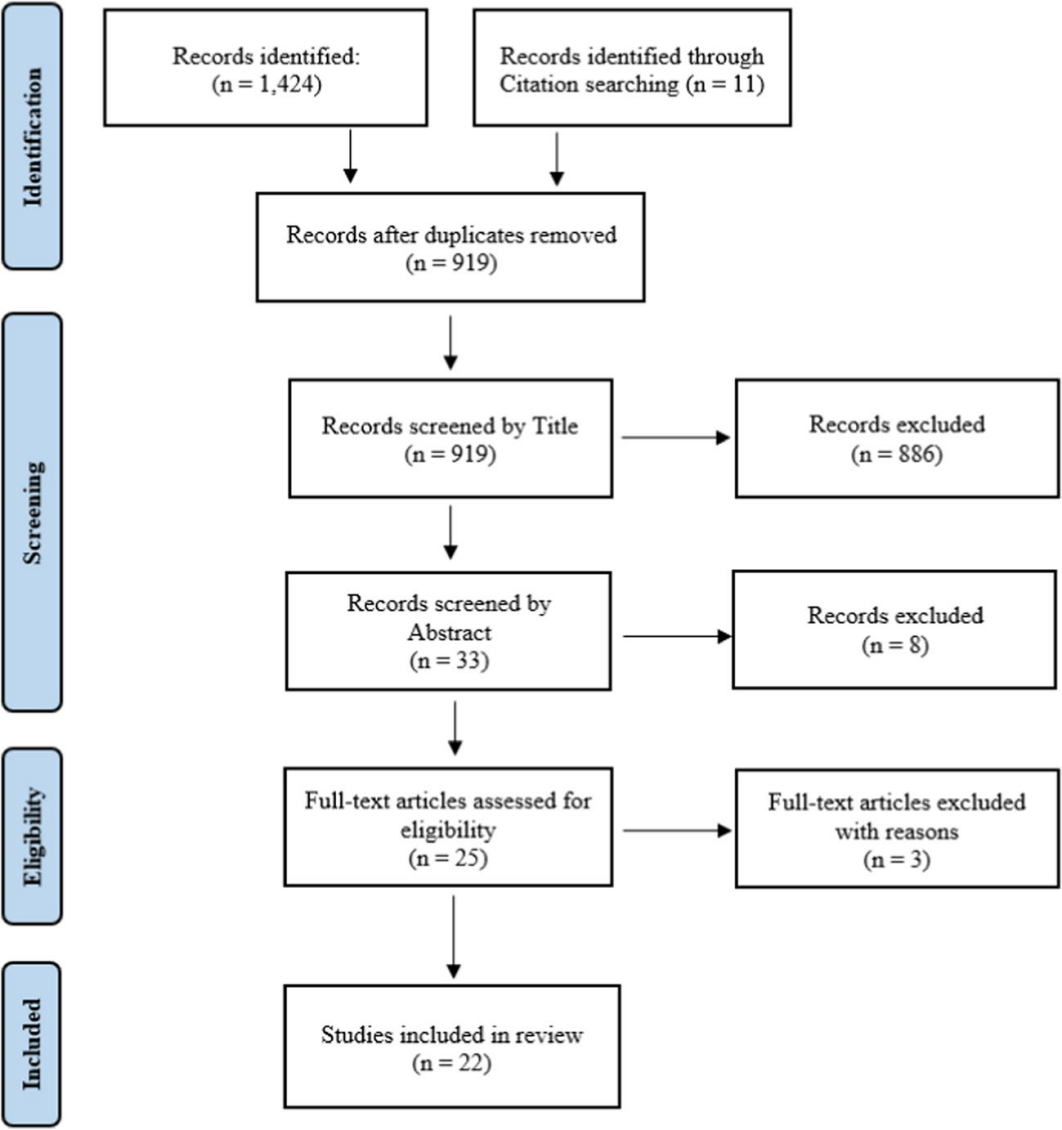
PRISMA flow diagram of scoping review process

Of the 918 unique articles found with the search strategy, 22 were selected as eligible for the scoping review. The selection process is documented in Fig. 1.

### Characteristics and results of sources of evidence relating to research question

Among the final 22 articles, all were peer-reviewed journal articles excepting one, which was a doctoral dissertation (Bothwell, 1985). The studies were spread over seven different countries, with 11 conducted in the USA (50%). There were three from the United Kingdom (13.6%). There were two each (9.1%) from Australia, Canada, and Slovenia, and one each (4.5%) from Sweden and Bosnia and Herzegovina. The research spanned 42 years in total, with the earliest study published in 1978 and the most recent in 2020. By decade, there were three from the 1970s (13.6%), three from the 1980s (13.6%), seven from the 1990s (31.8%), three in the 2000s (13.6%), four in the 2010s (18.2%), and three from the 2020s so far (13.6%). It is worth noting that 27.3% of these papers were published in the 10 years to 2022, indicating the current interest in this topic.

Of the studies, 17 included a participant sample of university/college students (77.3%), one (4.5%) drew participants from a university community (both staff and students), two from the general public (9.1%), one (4.5%) used existing witness statements from real criminal investigations, and one did not provide population information (4.5%). Nine articles (40.9%) did not provide information about participant age ranges or averages. Due to the high proportion of university/college samples, in those articles that did provide age-related information, the majority of the participant samples had a mean age that was below 27 years old. Regarding the methods used to assess eyewitness memory, 14 studies (63.6%) were recall-based, with free and/or cued recall tasks to assess accuracy (e.g., person, place, event details, etc.). There were five studies that included both recall and face-identification tasks (22.7%). There was one facial recognition study (4.5%), and one involving both facial recognition and face identification tasks (4.5%). There was also one study that used a working memory task (4.5%). Six of the studies (27.3%) also measured confidence in memory recall/face identification. Six studies (27.3%) did not provide a proposed explanation for the sex differences or similarities, and therefore do not contribute to answering research question 2. The information extracted from the articles is documented in Table 3.

**Table 3:** Information extracted from articles relevant to the three review questions (arranged in chronological order)

Author and year	Country	Participants	Research methods	Sex of central person/people	Sex differences	Sex similarities	Proposed explanations
Clifford and Scott 1978	England	- N = 48 - 24 males / 24 females - 18–30 years - Undergraduate students (non-psychology degrees)	- Two black-and-white videos of policemen searching for and finding a criminal (not shown) with the reluctant help of a third person - One involved a violent incident (1 min 4 s) - One involved a non-violent incident (1 min 3 s) - Half of participants (N = 24) in each condition - All participants completed Eysenck Personality Inventory (EPI) - 12 participants from each condition completed narrative, free recall of film’s content (particularly actions and descriptions) - All participants completed 44-item questionnaire assessing memory accuracy for physical actions and physical descriptions - Those in violent conditions rated how violent the video was (1 = slightly violent, 2 = very violent)	- Two male police officers - Third person also male	- Males were more accurate than females in the narrative recall condition - Females were more accurate than males in the interrogative report condition - Females were significantly less accurate than males overall in violent condition - Females rated the violent film as significantly more violent than males did - Females recalled actions better than descriptions in the violent condition - Males had better recall for descriptions than for actions in the violent condition	- Recall accuracy was generally low across conditions - However, recall was significantly less accurate for the violent incident - No overall difference in memory accuracy between males and females across conditions - Males and females did not differ overall in non-violent condition - Both males and females recalled actions more accurately than descriptions in the non-violent condition	- Females may be socialised to feel more fearful, vulnerable, and less capable than males in violent encounters
Powers et al. 1979	USA	Experiment 1: - N = 50 - 25 females /25 males - Undergraduate students	Experiment 1: - Series of 24 slides depicting a wallet snatching (Experiment 1) or a fight (Experiment 2). - A short filler task - 30-item multiple-choice test of accuracy for the person, event, and environment details - Confidence in accuracy was rated on a 3-point scale (1 = guessing, 3 = high confidence) - The following day, participants read a version of the incident that was either true or contained misleading information - 20-item test consisting of declarative sentences to complete with phrase or word from choices provided	Experiment 1: - Male perpetrator - Female victim	Experiment 1: - Own gender bias^a^ found for both males and females in accuracy and resistance to suggestion about own-gender oriented object details - Specifically, females were more accurate than males for details about women’s clothing or actions while males were more accurate for details about male thief’s appearance - Males were also more accurate than females for details about the surrounding environment - Females were more suggestible than males overall	Experiment 1: - No difference in accuracy overall between males and females - No difference in confidence ratings	- Suggest that males and females may not differ in accuracy overall, but that they recall different types of information Experiments 1 and 2 - The kinds of information they remember may be due to differential interest in particular items and therefore differential amounts of attention paid to those items
		Experiment 2: - N = 200 - 100 females / 100 males - Undergraduate students	Experiment 2: - A preliminary procedure involving 50 of the participants was conducted to identify separate sets of details from the slides likely to be noticed by males and females - A sequence of slides (one every 5 s) depicting a man and a woman witnessing a fight in a parking lot after which the man intervenes and the woman goes to a phone booth to call for help - Short filler task - 25-item accuracy questionnaire to assess memory for details related to central characters, clothing and actions, and surrounding environment, people, buildings - Following day participants read a version of the incident containing four pieces of misleading information - Final 18-item test consisting of declarative sentences with missing phrases or words to be completed with options from a provided list	Experiment 2: - One male and one female onlooker - Genders of two persons involved in the fight not specified	Experiment 2: - Own-gender bias found with females more accurate for female items and males more accurate for male items - Females were more suggestible than males for male-oriented items - Males were more suggestible for female-oriented items		
Yarmey and Jones 1983	Canada	- N = 48 - 24 males / 24 females - 16–44 years (m = 20.8)	- Sequence of 60 images (displayed 2 s each) depicting an assault and implied rape in a park - Verbal free recall task - 30 question multiple-choice questionnaire with 10 questions related to the victim, the rapist, and the environment - Photo line-up identification task to identify the rapist and then the victim - Confidence ratings for each identification - 21-item questionnaire assessing attitudes to rape	- Female victim - Male perpetrator	- Females had a greater tendency to report the rapist absent from photo line-up when he was present - Males had higher confidence in their identifications	- No significant sex differences for free recall, interrogatory recall, or identification of rapist or victim	- May be that criminal assault and rape scenario was equally salient to males and females. - Female witnesses may not have identified with or attended adequately to the female victim because they found the scenario too distressing
Bothwell 1985	USA	- N = 128 - 60 males / 68 females - Undergraduate students	- Participants exposed to target under low, moderate, or high arousal conditions: - Low: no item - Moderate: an empty hypodermic syringe package, small bottle of clear liquid, rubbing alcohol, cotton balls - High: same as moderate but with visible syringe - Pulse rates and scores on State Anxiety Scale to measure arousal - 21 open-ended questions about age, weight, height, facial features, and clothing of target person - 6-item photo line-up that either did or did not contain the target - Confidence judgements on 7-point Likert scale	- Male target person	- Males performed better than females at identifying target - Females performed better than males at rejecting line-up when target was absent - Females were less likely to choose than males and thus performed better for target-absent line-up as not choosing was correct response - Some evidence for same-sex bias i.e., males were better at identifying males but only in target-present condition	No reported sex similarities	No proposed explanation
Macleod and Shepherd 1986	UK	- 127 females / 252 males	- Data taken from 135 incidents occurring in Aberdeen during 1982 - All cases involved an assault, and had a single victim and single accused - Resulted in a total of 379 eyewitness statements.	- Unspecified	- Female witnesses gave significantly more details about themselves and the victim - Males provided more details about accused - Females reported significantly less information about the accused in cases where the victim sustained injury	- Males and females were similar for length of eyewitness statement	- Authors suggest that females may have lower capability to cope with stressful situations than males and thus have higher feelings of fear and vulnerability - Sex differences could be due to the allocation of cognitive resources – in real witness situations females may regulate perceived threat level by averting their gaze from the accused to avoid gaining their attention
Loftus et al. 1992	USA	- N = 1,989 - Children and adults (5 years and over) - 1,162 males / 827 females - Visitors to the Exploratorium museum	- A 1.25-min video of an assault at a political rally - 10 yes/no questions about details of the event and the scene of the event - Approx. half of participants got two misleading questions	- Male victim - Male perpetrator	- Males were more susceptible to misinformation effect	- No sex differences for recall accuracy	No proposed explanation
Yarmey 1993	Canada	- N = 651 - 348 females / 303 males - 18–65 years - Native English speakers - Drawn from general public	- Field study - One target person approached participants at random in public places and asked their assistance - 321 participants interacted with Target A and 330 with Target B - Eight prompted recall questions about the first target’s physical appearance - Estimated time of interaction - Confidence ratings on a 7-point Likert scale	- Two female targets	- Female participants had significantly higher recall accuracy for target’s weight - Trend towards significance for higher female accuracy in recalling hair colour and length.	- No differences between males and females for recalling other characteristics (eye colour, complexion, height, hairstyle, and age)	No proposed explanation
Shaw and Skolnick 1994	USA	- N = 191 - Authors state: half male / half female - Introductory psychology students	- Series of six images depicting a target person carrying an object through a scene - Six conditions – magazine (control), handgun (weapon), and four interesting / incongruous objects - Two questionnaires as distractor tasks - A recognition /identification task to identify the target person - 18-item recall questionnaire about the physical details of person	- One male or - One female	- Females identified the person more easily when the person was carrying a gun - Males were least accurate when space cones, gun and snake were present - Both males and females demonstrated own-gender bias in identification performance	No reported sex similarities	- For own-gender bias, superior identification may be result of inferential processing – Eyewitnesses may use inferential processing towards the same sex person (what kind of person are they?) but more superficial processing towards the opposite sex person (how attractive are they?) - Sex differences for object conditions may have been influenced by gender-orientation of the objects – males and females may have been more distracted by male and female oriented objects respectively
Butts et al. 1995	USA	- N = 40 - 20 males and 20 females - Undergraduate college students - 18–50 years (m = 26.2)	- Two stimuli images of a family at a dinner table (1) and vehicle accident in a busy street (2) - Two groups that viewed images in different order - 10-item questionnaire with both factual and leading questions	- Both males and females	No sex differences found.	- Males and females performed similarly in accuracy of recall and in resistance to misleading information	- There may be no sex differences to find - Differences between males and females may be changing and reducing due to socialisation patterns - It may not be a question of which sex is more accurate, but whether they are accurate in different ways
Casiere and Ashton 1996	USA	- N = 43 - 24 males / 19 females - College students	- Short video (3 min 50 s) of possible pickpocketing outside grocery store at night. - One open-ended free-recall question (“briefly describe what happened”) - Nine multiple-choice recall questions about the scene.	Unspecified	- Females were more accurate than males.	No reported sex similarities	No proposed explanation
Lindholm and Christianson 1998	Sweden	- N = 164 - 84 females / 80 males - Non-psychology university students	- Four groups for different victim/perpetrator sex pairings - A 4-min video of an implied manslaughter incident - A 10-min filler task - Free-recall task of the event - A questionnaire with eight rating items (emotional impact, responsibility of perpetrator, violence and provocativeness of the scene, etc.) - 25 open-ended questions about their recall of person details and event details.	- Male perpetrator / female victim - Male perpetrator / male victim - Female perpetrator / female victim - Female perpetrator / male victim	- Females were significantly more accurate overall in cued recall of the event, - Females were significantly more accurate for perpetrator descriptions particularly for female perpetrator in cued recall task - Females were also more accurate for recalling person details of female victim in cued recall task	- Males and females performed similarly for free recall	- Females may have a more elaborated set of cognitive categories relevant to person perception than males probably due to a greater female interest in this type of information. - Both males and females might also have more elaborated categories for same-gender than for opposite-gender targets. - May reflect a more general female advantage in episodic memory performance.
Shaw and Skolnick 1999	USA	- N = 200 - 100 male / 100 female - Undergraduate psychology students	- Short video depicting male/female intruder enters a lecture and confronts lecturer - 10 versions – half male / half female intruder and 5 object conditions (no object, a book, a gun, space cones, or a stethoscope) - Filler task - Questionnaire to test accuracy for person and object details	- One male or - One female	-Own-gender bias was observed for both males and females in control conditions (book object)	- No own-gender bias for weapon or unusual object - Own-sex intruder identification was lower for weapon and unusual objects - For opposite sex intruder accuracy for person details was higher for weapon or unusual object	- Carrying an interesting object may cause eyewitnesses to perceive a person of the opposite-sex as more attractive and interesting, thus leading the eyewitness to focus more attention on that person - When they are of the same sex interesting objects may only act as distractions, decreasing accuracy
Wright and Sladden 2003	UK	- N = 40 - 20 female / 20 male - 17–36 years (M = 26) - University students	- Images of 24 target faces, half with hair blacked out - Recognition task selecting target faces from 48 images - Confidence ratings out of 10 - Described recollection (remember, know, familiar, or guess)	- 24 male and 24 female faces	- Strong own-gender bias for both males and females, with hair a possible contributing factor - Hair was more helpful for own-gender identification than for other-gender identifications	No reported sex similarities	- Evolutionary / competition: more concerned with recognizing competition for mating than recognizing possible mates - In media such as magazines, majority of photographs are of people of the same gender as the target audience so people may develop better recognition for own-sex individuals
Sharps et al. 2007	USA	Study 1 - N = 198 - 149 females (M = 19.86) and 49 males (M = 21.10) - Psychology students Study 2 - N = 47 - 31 females (M = 25.68) - 16 males (M = 23.38) - College students	Study 1 - Series of images depicting the scene of a potentially violent crime with a male or female perpetrator holding a handgun or a hand-tool (e.g., screwdriver). - Two conditions – simple scene or complex scene - Police-style interviews to assess recall for person, weapon and scene details Study 2 - Scenes of male perpetrator from study 1 - Six-item photo line-up - ADHD and dissociative symptoms scales	Study 1 - One male perpetrator or - One female perpetrator Study 2 - Male perpetrator only	Study 1 - Females were better at describing the clothing of the perpetrator than males	Study 1 - No significant sex differences for memory of physical characteristics, weapon details, or peripheral sources of hazard Study 2 - No sex differences found in accuracy as only 5 out of 47 participants made accurate identifications	No proposed explanation
Pickel 2009	USA	Experiment 1 - N = 127 - 75% females / 25% males - Psychology students - 18–48 years (M = 19.31)	Experiment 1 - Video (1 min 30 s) depicting a perpetrator waiting in a car before jumping out to rob a male and female victim - Two object conditions with either a weapon or a neutral object (handgun/CD in plastic case) - Participants viewed one of four video versions - Written questionnaire asking questions related to the perpetrator, the object, and how threatening the perpetrator was	Experiments 1 - Both male and female victims across conditions - Either male or female perpetrator		- No significant sex differences found for either correct or incorrect details	- No proposed explanation
		Experiment 2 - N = 181 - 64% female / 36% males - Psychology students - 18–48 years (M = 19.69)	Experiment 2 - Same videos from the CD condition of experiment 1 - New videos with either a folding knife or knitting needle - Same procedure and questionnaire as Experiment 1	Experiment 2 - Same as used in Experiment 1		- No sex differences found	- No proposed explanation
		Experiment 3 - N = 255 - 60% female / 40% males - Psychology students - 18–39 years (M = 19.59)	Experiment 3 - 1-min video depicting a perpetrator stealthily walking through a hallway in a university building, entering a room where two students are studying and stealing a wallet off the table - Four versions of the video with perpetrator gender and object conditions - Two object conditions with either a weapon (switchblade knife) or a neutral object (highlighter pen) - Participants were primed with background on the perpetrator as either a violent and dangerous individual with a criminal record for robbery or a college psychology student	Experiment 3 - Different conditions with either a male or female perpetrator		- No sex differences found	- No proposed explanation
Areh 2011	Slovenia	- N = 280 - 161 females / 119 males - first-year undergraduate students - 18–21 years (M = 19)	- 2-min video of a violent robbery - 77-item feature checklist of recall for visual and audio event details - 7-point Likert scales to assess quality of memories and certainty in the memory	- Female victim - Male perpetrator	- Females were more accurate overall and reported fewer false details - Females were more accurate than males for person details, especially for the victim - Females were more accurate for place description - Small male advantage in describing the incident - Males had higher confidence in the reliability of their memory	- Excluding accuracy person details, differences in other recall were small or non-existent - Quantity of memory recall was the same for males and females	- Females may be better at describing other females because they may pay more attention to them - Females may have identified with female victim – i.e., more motivated to be detailed and accurate
Palmer et al. 2013	Australia	Study 1 - N = 113 - 68 females / 45 males - 17–39 years (M = 19.7) - Undergraduate students Study 2 - N = 502 - 280 females / 222 males - 17–56 years (M = 22.4) - Undergraduate students	Study 1 - Two blocks of facial recognition trials. - One block under full attention conditions and one block under divided attention conditions, Study 2 - Data re-analysed from a previous study - Participants viewed one male and one female culprit and identified each culprit from a separate line-up	- Both males and females	Study 1 - Divided attention at encoding reduced the female own-gender bias - No own-gender bias effect found for male participants for either full or divided attention conditions Study 2 - Own-gender bias present for both males and females	Study 2 - Own-gender bias reduced in divided attention conditions - Other-gender recognition did not differ between conditions for males or females	- Female own-gender bias relies on attention at encoding (at least in part) - Females pay more attention to female faces than male faces for social or developmental reasons
Zoladz et al. 2014	USA	- N = 60 - 30 males / 30 females - University students - Mean age = 19.18 years	- Stress condition - dominant hand submerged in an ice bath (3 min) - No stress condition - sat quietly (3 min) - Stress rated on 11-point scale - Working memory task	NA	- Stress enhanced true memory recall and recognition for females but no effect for males		- Females may have exhibited better memory accuracy because of a greater attention to detail (possibly because of greater left amygdala activation)
Horgan et al. 2017	USA	- N = 508 - 266 Females / 242 Males - Introductory psychology undergraduate students	- Four groups - 3- to 5-min video of target person introducing themselves (different individuals for each group) -Yes-no/multiple-choice questionnaire about the physical features and dress-related items	- Two females - One male	- Females were more accurate at recalling what the target was wearing - Own-gender bias was stronger for female target - Both males and females showed own-gender bias	- Males and females performed similarly for recalling physical features	- Authors argue that clothing/accessories is more of a female domain of interest as they spend significantly more time learning about clothing and accessories (what is in fashion, what is flattering etc.) from a young age - Heterosexual females compete with other females for males, and thus may have vested interest in the clothing/accessories of other women for reasons of social comparison (authors note that this does not explain higher accuracy for men’s clothing/accessories)
Areh and Walsh 2020	Slovenia	- N = 256 - 135 females / 121 males - First-year undergraduate students - 18–21 years (M = 19)	- Four groups with different victim/perpetrator sex pairings - Two-minute video recording showing a mock assault and robbery - Free recall account of the event - 28 multiple-choice questions about perpetrator.	- Male perpetrator / female victim - Male perpetrator / male victim - Female perpetrator / female victim - Female perpetrator / male victim	- Own-gender bias demonstrated with males more accurate for details of male perpetrator and females more accurate for details of female perpetrator	-Poor accuracy for personal descriptions overall - No sex differences in number of personal descriptors recalled for either the male or female perpetrators.	
Fazlic et al. 2020	Bosnia and Herzeg-ovina	- N = 98 - 55 males / 43 females - Undergraduate university students - Mean age = 18.9 years	- 44-s video of simulated bank robbery - 5-min distractor task either describing perpetrator or listing European capital cities - 20-min crossword puzzle distractor task - Identification task to identify perpetrator from 8-item photo line-up	- Male perpetrator		- Males and females performed similarly for identification accuracy - Males and females made mistaken identifications at similar rates - No own-gender bias found for males (relevant to males only as the perpetrator was male)	- Gender of eyewitness may not be a useful predictor of accuracy by itself, but may interact with other combined factors such as age, race, culture – particularly regarding social stereotypes about gender differences
Longstaff and Belz 2020	Australia	- N = 115 - 77 females, mean age = 39.7 years 38 males, mean age = 40.5 years - University students	- Narrative text that primed the scenario - POV video (1 min, 43 s) of a person walking through a hallway and unexpectedly meeting a stranger (section 1), who is revealed as female or male (section 2) - Multiple-choice questions for recall of environmental, object, and person details - Likert scales to rate feeling about the scenario (i.e., anxiety and threat)	- One male or - One female	- Females had higher accuracy for stranger-related questions - Females were more accurate at identifying sex of stranger - Females had higher levels of anxiety and viewed stranger as more threatening - Males were slightly more accurate for surroundings-related questions	- Males and females performed similarly for total accuracy and general questions - Both males and females demonstrated a bias towards identifying the stranger as male	- Suggest sex differences in accuracy and sex identification may occur because females focus attention on the stranger relatively longer out of caution and because processing person information is an evolved adaptation for females - Overall bias to identify stranger as male could provide a functional evolutionary benefit (e.g., safety) - Higher anxiety and perceptions of stranger threat led female participants to focus on the stranger, thus resulting in better memory for the stranger

^a^The own-gender bias is referred to as a sex difference in these articles, as male participants demonstrate better memory for males, and female participants demonstrate better memory for females. However, it is worth noting that in this case, this could also be characterised as a sex similarity in that they are also showing a similar memory bias towards individuals of their own gender

## Discussion

The present scoping review aimed to review the existing literature in order to investigate whether there are sex differences in eyewitness memory, what these differences may be, how they have been studied, and what explanations have been proposed for any differences found. A total of 22 primary research studies from seven countries and spanning a 42-year period (with six in the 10-year period up to 2022) were found and examined to answer these questions.

Not all of the studies compared males and females for recall, recognition and/or identification accuracy overall, and findings differed between those that did. However, some trends did emerge. Interestingly, although there was a tendency for males to be significantly more confident in the accuracy of their recall than females, none of the studies found males to be more accurate overall (Areh, 2011; Yarmey & Jones, 1983). On the contrary, three studies found that females had significantly more accurate recall overall and recalled fewer false details (Areh, 2011; Casiere & Ashton, 1996; Zoladz et al., 2014). Lindholm and Christianson (1998), however, found that this female superiority was only evident for cued recall, while the advantage disappeared for free recall. Nonetheless, it was suggested that the higher accuracy demonstrated by females may reflect a more general superiority in episodic memory recall (Lindholm & Christianson, 1998). The remaining studies that measured overall accuracy for recall (Butts et al., 1995; Clifford & Scott, 1978; Loftus et al., 1992; Longstaff & Belz, 2020), face identification (Fazlic et al., 2020), and both recall and face identification (Sharps et al., 2007; Yarmey & Jones, 1983) all found that there were no differences between males and females. It should be noted, however, that Sharps et al. (2007) state that in their study this was due to the fact that there were too few participants in total who made accurate identifications.

Although Butts et al. (1995) suggested that the absence of sex differences overall may indicate that there are no differences to find, the more common consensus was that sex differences may instead lie in the type of information accurately recalled (Areh, 2011; Butts et al., 1995; Powers et al., 1979). Furthermore, studies that compared the quantity of information and number of details recalled consistently demonstrated that there were no differences between males and females (Areh, 2011; Areh & Walsh, 2020; MacLeod & Shepherd, 1986). This suggests that any potential sex differences are not due to differences in the amount of information males and females are each able to recall.

While they may not have found any differences in overall accuracy, many studies did find specific differences in accuracy for specific kinds of information. One fairly consistent finding was that females were significantly more accurate in recalling person-related details, which typically included details about age, height, clothing, hair, and facial features (Areh, 2011; Lindholm & Christianson, 1998; Longstaff & Belz, 2020). This was true even in studies where recall was measured for both a perpetrator and a victim (Areh, 2011; Lindholm & Christianson, 1998). Furthermore, females were also more accurate at identifying the sex of a stranger when this was deliberately kept ambiguous (Longstaff & Belz, 2020). There was also a female advantage for describing and recalling details related to clothing (Horgan et al., 2017; Sharps et al., 2007) and a person’s weight (Yarmey, 1993). However, recall for other general physical features, including eye colour, height, age, and hairstyle, did not differ between males and females (Horgan et al., 2017; Sharps et al., 2007; Yarmey, 1993). Performance for event details has been less consistent, with some studies finding females to be more accurate (Lindholm & Christianson, 1998), and others finding a male advantage (Areh, 2011). Males have also been found to demonstrate greater recall for details related to the surroundings, although once again the difference was small (Longstaff & Belz, 2020).

One study also found that males were more susceptible to the misinformation effect (Loftus et al., 1992), although this was contradicted by a second study, which found that there were no sex differences in resistance to false information (Butts et al., 1995). Overall, the findings seem to indicate that sex differences in eyewitness memory are not a question of whether males or females are more accurate in general, but instead reflect specific differences in the types of information that are recalled more accurately by each.

The most consistent finding to emerge was the own-gender bias effect. Participants were consistently more accurate when recalling details for a person of their own gender and also increased accuracy for recognising and identifying a target person (Areh & Walsh, 2020; Longstaff & Belz, 2020; Palmer et al., 2013; Powers et al., 1979; Shaw & Skolnick, 1994; Shaw & Skolnick, 1999; Wright & Sladden, 2003). Furthermore, participants were also more resistant to suggestion when recalling own-gender details (Powers et al., 1979). While this own-gender bias was common and consistent, some findings suggest that its presence, and the strength of the effect are contingent on other factors. For example, Wright and Sladden (2003) argued that encoding information about a target person’s hair accounts for a significant portion of the own gender bias, proving more useful when making own-sex identification than opposite-sex identification. On the other hand, divided attention during encoding was found to reduce the own-gender bias (Palmer et al., 2013). Furthermore, the presence of specific objects may reverse this effect, with one study finding that accuracy for opposite-sex identification was significantly higher when the person carried a weapon or an unusual object (Shaw & Skolnick, 1999).

Several different suggestions were made to explain why males and females may differ in the types of information they recall. The most common consensus was that these differences result from differences in attentional focus, with males and females attending to different stimuli due to varying levels of interest (MacLeod & Shepherd, 1986; Powers et al., 1979). For instance, one study suggested that the female superiority for accurately recalling person-related details occurs because females have higher level of interest in this type of information and have thus developed more elaborate cognitive categories for it (Lindholm & Christianson, 1998). Similarly, Horgan et al. (2017), suggested that females perform better for recalling clothing and accessories because it is a gendered domain of interest and so more attention is focused to those details.

Perceived threat was another factor that was suggested to direct female attention to person information. Longstaff and Belz (2020) found that females reported higher levels of anxiety and perceived threat, and argued that they may therefore have focused more attention on the stranger out of caution, resulting in better recall for person details. Differences in attentional focus was also the main explanation provided for the own-gender bias effect. One study found that attention during encoding is responsible for a significant portion of this effect (particularly for females), with males and females paying significantly more attention to own-gender faces (Palmer et al., 2013). Some authors suggested a social influence for this bias, arguing that people develop better own-sex recognition because they consume media targeted towards their own gender, which generally contains more own-gender images (Wright & Sladden, 2003). It has also been argued that evolutionary factors direct a person’s attention towards other of their own sex for purposes of social comparison, as there is evolutionary benefit in recognising competition for mating (Horgan et al., 2017; Wright & Sladden, 2003).

While many of the studies suggested possible explanations for the differences found between males and females, the majority of these were based on post-hoc theorising. As such, no measures were included in these studies (with the exception of Longstaff & Belz, 2020) to test the explanations proposed. This presents an issue when attempting to answer the second review question. It also highlights a significant gap in the literature, as there is still a lack of sound, evidence-based theories to explain why sex differences in eyewitness memory may occur. Given that the findings of this review indicate that sex differences do exist for specific types of information, clarifying the reasons for these effects is of importance. Future research in this area should therefore focus on designing methodologies that are able to empirically test theoretical explanations.

Through our review of these articles, it is clear that there is significant variation in the way that the research has been conducted. This is not surprising, given that the topic of eyewitness memory is such a broad one. Most of the studies included in this review measured eyewitness memory by creating mock eyewitness scenarios in videos or a series of images and then measured accuracy for details related to those scenes. However, even for those studies with broadly consistent methodologies, the content of these scenes varied, ranging from violent crimes (robbery, manslaughter, assault/rape) to ambiguous scenes, and even to innocuous scenes such as male and female targets introducing themselves. The methods used to measure memory also differed between articles. For instance, while some studies measured recall accuracy using free recall tasks, others used cued recall questionnaires or checklists, and some used a combination of these methods. Furthermore, it must be noted that while the studies referred to multiple-choice questions and checklists as cued recall tasks, these are technically recognition tasks. The lack of consistency in the methodologies and terminology used between studies reflects the complexity of eyewitness memory as a topic for research, and may help to explain the lack of consensus regarding sex or gender differences across the literature.

The diverse methodologies also make it difficult to definitively conclude whether there are sex differences in eyewitness memory and what the differences may be. Given that the literature on sex differences is limited compared to other topics in eyewitness memory research, this variability in both methodology and terminology presents issues when it comes to comparing and generalising findings between studies. Therefore, further research with more consistent methodologies is needed to develop a reliable foundation within the literature and also to develop a more systematic approach to answering the question, or aspects thereof.

The research reviewed here also spans more than 40 years. Eyewitness memory research, and the field of cognitive psychology more broadly, has developed significantly during this time, and so too has research on sex and gender. This presents potential difficulties with comparing the findings of the earlier studies to those of more recent research. The majority of the studies drew their participant samples from university or college cohorts, which also makes it difficult to generalise the findings to the broader public. Furthermore, the vast majority of the included articles were from developed countries, with half of the research conducted and published in the USA. This lack of cultural diversity provides little opportunity to gain insight into how these findings compare across different countries and cultures. Furthermore, many of the studies reviewed suggested social influences as a factor that may explain how and why males and females may differ in some aspects of eyewitness memory. Therefore, it is relevant to gain a better understanding of how cultural differences in social stereotypes surrounding gender may influence sex differences in eyewitness memory, particularly as many of these western/developed countries become increasingly multicultural.

Our scoping review also had some limitations. While we sought to examine sex differences in eyewitness memory, it must be noted that all the studies reviewed relied on self-reported sex/gender. Furthermore, the terms ‘sex’ and ‘gender’ were used interchangeably through most of the studies, all of which seem to have assumed both terms to refer to sex and gender as the biological variable (male or female). When extracting data and discussing the studies, we have therefore used the terms as they were used in each article. Furthermore, the language limiter used in the search strategy may mean that some relevant articles were excluded. Nevertheless, the number of search results in languages other than English were very low, indicating that the majority of relevant results were captured by the search strategy.

There are also limitations inherent to the screening processes employed when conducting scoping (and other) reviews. It is possible that there were some studies captured by the initial search strategy that do contain some relevant findings or analyses related to sex or gender differences in eyewitness memory but where this information was not mentioned in the title or abstract. For example, these findings might be incidental to the main focus of the study and only briefly noted in the body text. In these cases, following the PRISMA guidelines for scoping reviews, the full text would not be read and this information would be missed. These then end up being excluded during the initial stages of the screening process when examining the titles and abstracts. The screening process is both time and labour-intensive and therefore it is not feasible to examine the full texts for every article captured by the original database searches.

## Conclusion

This scoping review is the first such review of the literature related to sex differences in eyewitness memory. Although this topic has maintained sustained research interest and been subject to investigation since at least the 1970s, the literature is limited and lacks consensus. However, despite the variability in methodologies and findings, some interesting trends emerge. Firstly, findings from the studies reviewed here suggest that neither males nor females have a clear advantage for accuracy overall, but that they may instead be more accurate for different types of information. There was a tendency for females to demonstrate significantly higher accuracy for person-related memory, perhaps due to differential interest and attention in that type of information. There was also some evidence that males had a slight advantage for details related to the surrounding environment. The most consistent, though not universal, finding was that both males and females typically perform better in identifying, recognising, and recalling details related to a person of their own gender. This own-gender bias was suggested to be the result of people focusing greater attention on members of their own sex due to social and/or evolutionary factors. Overall, the diversity of findings related to the diversity of methodologies may indicate that any differences between males and females are context and task specific. Although there was some consensus for the proposed attentional component for these sex differences, no compelling causal information or evidence was provided for why differences may occur. Given the ongoing scientific, social, and political relevance of this topic, future research should seek to clarify if and how attentional focus may differ between males and females, and how this translates to differences in eyewitness memory.

## Data Availability

Not applicable.

## Appendix

**Table 4:** Search strategy for APA PsycInfo via EBSCO database

Step	Search Terms	Results	Date
1	DE “Sex”	4,489	25/04/2022
2	sex* or gender or m?n or wom?n or male or female or boys or girls	1,892,814	25/04/2022
3	sex* or gender or m?n or wom?n or male or female	1,859,962	25/04/2022
4	S1 OR S3	1,859,962	25/04/2022
5	eyewitness*	3,549	25/04/2022
6	DE “memory”	93,062	25/04/2022
7	“memory recall” or “memory retrieval” or “memory retention” or “memory reconstruction”	60,106	25/04/2022
8	S6 OR S7	96,440	25/04/2022
9	experiment* OR study OR studies	2,895,178	25/04/2022
10	AB experiment* OR study OR studies	2,397,568	25/04/2022
11	S4 AND S5 AND S8 AND S10	227	25/04/2022
12	S4 AND S5 AND S8	351	13/05/2022
13	Narrowed by language (English)	336	13/05/2022
